# Monogenic Autoinflammatory Syndromes: State of the Art on Genetic, Clinical, and Therapeutic Issues

**DOI:** 10.1155/2013/513782

**Published:** 2013-10-24

**Authors:** Francesco Caso, Donato Rigante, Antonio Vitale, Orso Maria Lucherini, Luisa Costa, Mariangela Atteno, Adele Compagnone, Paolo Caso, Bruno Frediani, Mauro Galeazzi, Leonardo Punzi, Luca Cantarini

**Affiliations:** ^1^Rheumatology Unit, Department of Clinical and Experimental Medicine, University of Padua, Padova, Italy; ^2^Institute of Pediatrics, Università Cattolica Sacro Cuore, Rome, Italy; ^3^Interdepartmental Research Center of Systemic Autoimmune and Autoinflammatory Diseases, Rheumatology Unit, Policlinico Le Scotte, University of Siena, 53100 Siena, Italy; ^4^Rheumatology Unit, Department of Clinical and Experimental Medicine, University Federico II, Naples, Italy; ^5^University La Sapienza, Rome, Italy

## Abstract

Monogenic autoinflammatory syndromes (MAISs) are caused by innate immune system dysregulation leading to aberrant inflammasome activation and episodes of fever and involvement of skin, serous membranes, eyes, joints, gastrointestinal tract, and nervous system, predominantly with a childhood onset. To date, there are twelve known MAISs: familial Mediterranean fever, tumor necrosis factor receptor-associated periodic syndrome, familial cold urticaria syndrome, Muckle-Wells syndrome, CINCA syndrome, mevalonate kinase deficiency, NLRP12-associated autoinflammatory disorder, Blau syndrome, early-onset sarcoidosis, PAPA syndrome, Majeed syndrome, and deficiency of the interleukin-1 receptor antagonist. Each of these conditions may manifest itself with more or less severe inflammatory symptoms of variable duration and frequency, associated with findings of increased inflammatory parameters in laboratory investigation. The purpose of this paper is to describe the main genetic, clinical, and therapeutic aspects of MAISs and their most recent classification with the ultimate goal of increasing awareness of autoinflammation among various internal medicine specialists.

## 1. Introduction

In the recent years, the identification of genes involved in the modulation of inflammatory and apoptotic processes and the improved understanding of mechanisms linked to the aberrant activation of the inflammasome, amultiprotein intracytoplasmatic scaffold complex synthesizing the biologically active interleukin- (IL-1), the prototypic master cytokine affecting nearly all cell types, have allowed the delineation of a new group of diseases called “monogenic autoinflammatory syndromes (MAISs)” [1].

From the etiopathogenetic point of view, in spite of the heterogeneity of genes responsible for the various MAISs (Table 1), the inflammasome represents an ideal point of convergence of most of these diseases, that is, the cell structure crucial to the regulation of innate immunity: its proper assembly allows for regular activation of caspase-1 and physiological production of proinflammatory cytokines, *in primis* IL-1*β*, necessary to respond to a heap of different danger signals, as bacterial peptidoglycans, genotoxic stress, and crystals. In the pathogenesis of many MAISs, the erroneous assembly of the inflammasome leads to an exaggerated conversion of pro-IL-1*β* to its active form and subsequent disproportionate overwhelming inflammatory response [2].

The term “autoinflammatory,” used in contrast to the term “autoimmune,” was intended to highlight the spontaneous nature of the inflammatory attacks, which occur in the absence of any pathogenetic role of autoantibodies or autoreactive T lymphocytes. Therefore, the contribution of as-yet unidentified environmental factors as potential triggers of abnormal inflammatory processes might be likely [3, 4]. Clinically speaking, a few characteristics common to all MAISs have been identified, such as the recurrent nature of inflammatory episodes, presence of fever, and frequent involvement of the skin, serous membranes, eyes, joints, lymph nodes, gastrointestinal tract, and nervous system. Each of these syndromes may manifest itself with more or less severe inflammatory signs and symptoms of varying frequency and duration, associated, from the laboratory point of view, with increased phlogistic parameters [5, 6] (Table 2).

To date, there are twelve known MAISs: familial Mediterranean fever (FMF); tumor necrosis factor receptor-associated periodic syndrome (TRAPS); cryopyrin-associated periodic syndrome (CAPS), a group which includes familial cold urticaria syndrome (FCAS), Muckle-Wells syndrome (MWS), and chronic infantile neurological cutaneous articular (CINCA) syndrome; mevalonate kinase deficiency (MKD); NLRP12-associated autoinflammatory disorder (NLRP12AD); granulomatous MAISs which include Blau syndrome (BS) and early-onset sarcoidosis (EOS); and, finally, the hereditary pyogenic disorders including PAPA (pyogenic arthritis, pyoderma gangrenosum, and acne) syndrome, Majeed syndrome (MS), and deficiency of the IL-1 receptor antagonist (DIRA).

MAISs are generally characterized by early onset (in the first year of life or early childhood) [4], but, in more than a few cases, in particular for FMF and TRAPS, adult onset has also been described [7, 8]. In such cases, the utilization of a highly sensitive and specific score can be useful in guiding diagnosis [9–11]. Type AA amyloidosis is the most serious complication of most MAISs, due to excessive production of serum amyloid-A (SAA), synthesized in the liver following stimulation by certain proinflammatory cytokines, such as IL-1*β*, and also IL-6 and tumor necrosis factor-*α* (TNF-*α*). Due to persistent activation of the chronic inflammatory process, whether clinically manifested orsubclinically, excess SAA is deposited in the form of fibrils in various organs, particularly the kidneys, with the consequent progressive development of severe proteinuria, leading to nephrotic syndrome and kidney failure. Other areas that may be involved include the autonomous nervous system (with orthostatic hypotension, impotence, and altered intestinal motility), liver and spleen (with hepatosplenomegaly), muscles, heart (with contractility and circulation abnormalities), and gastrointestinal tube (with diarrhea and malabsorption). Therefore, close monitoring of serum SAA levels even during healthy periods is necessary to prevent or promptly treat a secondary amyloidosis or to verify the efficacy of treatment [12].

From a therapeutic point of view, colchicine has been proven to be the treatment of choice for patients with FMF [13], while nonsteroidal anti-inflammatory drugs (NSAIDs) and corticosteroids are utilized above all to treat symptoms in most of the other MAISs, with varying results. The introduction of biological agents, such as anti-TNF (etanercept, infliximab, and adalimumab) and anti-IL-1 (anakinra, canakinumab, and rilonacept), has nonetheless opened up new interesting possibilities for the management of these heterogeneous disorders [14].

The purpose of this paper is to describe the main genetic, clinical, and therapeutic aspects of MAISs, focusing on their current classification and general details, shown in Tables 1 and 2, with the ultimate goal of increasing awareness of these conditions among various specialties of internal medicine. 

## 2. Familial Mediterranean Fever (FMF)

Familial Mediterranean fever (OMIM 249100) is transmitted by autosomal recessive inheritance and, in its most frequent and classic phenotype, is characterized by recurrent acute fever episodes, polyserositis, arthritis, and erysipelas-like erythema [15]: it is due to the presence of mutations (among the 200 identified to date) in the *MEFV* (from MEditerranean FeVer) gene which encodes the protein pyrin, also known by its European name “marenostrin” [16, 17] (Table 1). This protein is made up of 781 amino acids and is expressed mainly in neutrophil and eosinophil granulocytes, monocytes/macrophages, and fibroblasts of the skin, peritoneum, and synovia. Pyrin mutations cause altered inflammasome function, which leads to increased synthesis of proinflammatory cytokines (mainly IL-1*β*), activation of transcription factor NF-*κ*B, and altered inhibition of apoptosis, all demonstrated in subjects with FMF [17–20].

In the past, FMF was believed to pertain almost exclusively to populations living near the Mediterranean basin, but today it is widely held that other populations can also be affected. Still, the most affected populations continue to be Armenians, Turks, Arabs, and non-Ashkenazi Jews with a rate of occurrence that oscillates between 1 : 400 and 1 : 1.000 in Turkey and that is around 1 : 1000 in Israel and 1 : 500 in Armenia [21]. The male/female ratio is 2 : 1 [22]. Onset of FMF usually occurs in the first two decades of life, with a relatively small number of adult-onset cases [7, 8]. 

From the clinical point of view, three different FMF phenotypes have been identified [23]: phenotype 1 is characterized by recurrent inflammatory episodes lasting 12–72 hours, sometimes triggered by stress, physical exercise, or infections and preceded by nonspecific symptoms like lack of appetite, malaise, and irritability. During these episodes, fever and abdominal pain are the most frequent clinical manifestations and may occur alone or in tandem. Abdominal pain due to sterile peritonitis may sometimes last a few days after the fever has ceased. About half of patients present thoracic pain with acute pleuritis, almost always monolateral, and/or pericarditis [15, 24]. Inflammation of the tunica vaginalis of the testis may also occur, leading to recurrent episodes of acute orchiepididymitis in these subjects [25].

Skin manifestations, also of brief duration (usually 12–48 hours) and generally associated with fever, are characterized by the periodic appearance of erysipelas-like lesions, approximately 10 cm in diameter, usually localized on the surface of the legs between the hip and knee and/or on the tops of the feet [26].

Muscular-skeletal involvement is frequent, often in the form of arthralgia and myalgia, which may be prolonged and crippling, significantly reducing patients' quality of life [27, 28]. About 30% of FMF patients have also arthritis, especially affecting the large joints, which may last for several days after fever has resolved: arthritis is only rarely erosive and is generally mono- or oligoarticular [27]. Possible, but rare, is aseptic meningitis, accompanied by headache and possibly by electroencephalographic alterations and convulsions [29]. In addition, FMF has also been associated with other rheumatological diseases, such as spondyloarthritis [30], rheumatoid arthritis [31], systemic lupus erythematosus [32], vasculitis of small and medium vessels (like Henoch-Schöenlein purpura, polyarteritis nodosa, and Behçet's disease), and vasculitis of large vessels (like Takayasu's arteritis) [33]. Most FMF patients enjoy good health from a clinical standpoint between acute episodes [34].

Acute episodes are associated with increased laboratory phlogosis indicators, particularly erythrosedimentation rate (ESR), C-reactive protein (CRP), SAA, and fibrinogen; other laboratory findings may include neutrophil leukocytosis, thrombocytosis, anemia, and, less frequently, an increase in immunoglobulins, particularly classes A and D [6].

The increase in SAA during FMF attacks, which is also possible during asymptomatic periods, is a clue to the progression towards amyloidosis: SAA measurement is thus a useful parameter for highlighting a state of subclinical inflammation and revealing a potential secondary systemic amyloidosis [6, 12, 35].

Phenotype 2 refers to FMF patients with proteinuria or kidney failure resulting from amyloidosis, in whom the inflammatory attacks typical of FMF occur only afterwards. This phenotype also includes subjects belonging to the families of FMF patients who evolve towards systemic amyloidosis as the sole manifestation of the disease [36–38]. Phenotype 3 includes subjects carrying one of two mutations (homozygous or heterozygous) of the *MEFV* gene without presenting with any of the known clinical manifestations [39].

Diagnosis of FMF is primarily clinical and based on the use of the Tel-Hashomer diagnostic criteria, divided into major and minor signs, as shown in Table 2: the presence of two major criteria, or one major and two minor criteria, allows for a definitive FMF diagnosis, while the presence of a single major criterion and one minor one may point towards a probable diagnosis, which can be confirmed thereafter by the presence of mutations in the *MEFV* gene [40]. The most common of the *MEFV* gene mutations is M694V (in exon 10), which, in homozygous cases, is correlated with an earlier disease onset and, more frequent, joint involvement, and occurrence of amyloidosis [41, 42].

From a therapeutic point of view, colchicine is now recognized as the drug of choice in the treatment of FMF, as it is effective in almost 95% and completely prevents the acute episodes in 60% of the patients. In addition, colchicine has also been proven to be effective in preventing secondary complications of amyloidosis [13, 14, 43–48]. Unlike the case of gout, colchicine is not effective in aborting an established acute episode and should be used for prophylaxis only. Initial dose is usually 1–1.2 mg daily, to increase every 1-2 months (depending on the frequency of the acute attacks) until an effective response is obtained, up to a maximal dose of 2.0–2.4 mg per day, if tolerated. Optimal dosage should be determined on case-by-case basis to achieve maximal efficacy with minimal side effects. In children with FMF, 0.02-0.03 mg/kg/day of colchicine should be given, up to a maximum daily dosage of 1.8–2.0 mg/day. Since colchicine treatment is often complicated by frequent gastrointestinal side effects, some experts recommend lactose-free diet in order to improve colchicine tolerance [49]. Colchicine therapy for FMF during pregnancy has not been reported to harm either the mother or her fetus [50]. Contraindications to colchicine will include hypersensitivity to any component of its formulation and severe renal or hepatic impairments, requiring a cautious use in the elderly with renal, liver, or biliary disease. NSAIDs and corticosteroids, sometimes at high doses, rarely achieve satisfying clinical results to control the disease [14, 51, 52]. Valid therapeutic alternatives in patients who fail to respond to colchicine include IL-1 inhibitors (anakinra, canakinumab, and rilonacept) [53] and anti-TNF-*α* agents (adalimumab, etanercept, and infliximab) [54].

## 3. TNF Receptor-Associated Periodic Syndrome (TRAPS)

TRAPS (OMIM 142680) is an autosomal dominant disease caused by prevalently missense mutations in the *TNFRSF1A* gene, made up of 10 exons encoding for the p55 1A receptor of TNF (TNFR1A): the vast majority of mutations are found on exons 2, 3, 4, and 6 [16, 55, 56], and they can be distinguished as high- or low-penetrance ones. The former are located in cysteine-rich N-terminal domains, fundamental for the assembly of the receptor's three-dimensional structure [57, 58], and they are characterized by early disease onset and more severe clinical manifestations; the low-penetrance mutations, such as R92Q and P46L, tend to be associated with onset of disease in adulthood and less pronounced or atypical clinical characteristics [59–65].

Although the biological alteration involves the TNF receptor, the pathogenesis of TRAPS also seems to be associated with a dysregulation in the secretion of IL-1 and IL-6, as well as oxidative damage correlated with the mitochondrial production of free radicals [61, 66, 67]. 

Clinically speaking (Table 2), patients complain of inflammatory attacks of extremely variable duration and intensity (from 1-2 days to 3-4 weeks), characterized by fever episodes accompanied more or less constantly by sterile peritonitis with abdominal pain, diarrhea or constipation, nausea, and vomiting [55, 68, 69]. Mono- or bilateral periorbital edema is a very characteristic and almost pathognomonic sign of the disease, often associated with conjunctivitis and periorbital pain [56]. Also very frequent are arthralgias, muscle cramps, and/or centrifugally spreading migratory myalgias and chronic fasciitis. Muscular symptoms may include edema and swelling of the muscular group involved, usually localized [68], while skin symptoms mostly include a serpiginous rash consisting of migratory and painful patches, histologically characterized by the presence of perivascular lymphocytic and monocytic infiltrates [70, 71].

Serous membrane inflammation is also common, usually in the form of polyserositis [62–65, 72–74]. Pericardial or myocardial involvement has also been reported as the only clinical manifestation of TRAPS [9–11, 62, 64, 71, 73, 75–77]. 

During acute episodes, and sometimes also in asymptomatic periods, there is a marked increase in phlogosis indicators (ESR, CRP, and SAA), as well as neutrophil leukocytes, aptoglobin, fibrinogen, and platelets [5, 6]. In up to 25% of patients carrying mutations involving cysteine residues and in about 2% of those carrying low-penetrance mutations, the emergence of secondary amyloidosis should be kept in mind. Therefore, proteinuria and SAA serum levels must be constantly monitored to avoid overlooking an occult subclinical amyloidosis and its progression towards end-stage kidney damage, which is the most dreaded complication of the disease [56, 59].

Diagnosis requires the identification of a mutation in the *TNFRSF1A*: thus, for patients with clinical symptoms that lead to the suspicion of TRAPS, genetic tests are indispensible. 

From a therapeutic point of view, high doses of NSAIDs and corticosteroids may prove useful during acute phases, though they do not reduce the frequency of attacks and furthermore do not prevent amyloidosis. In addition, when administered for long periods of time, high-dose corticosteroids can cause serious systemic side effects. Colchicine and immunomodulating or immunosuppressant agents have also been proven to have very little efficacy in TRAPS [14, 55, 68, 72, 78].

Due to the genetic defect at the origin of the pathology, it was clear that the use of anti-TNF agents could have an important effect in these patients. In fact, etanercept has been proven to be useful in reducing the intensity and duration of acute attacks, although in some cases it gradually loses efficacy [78–81]. Infliximab and adalimumab, by contrast, for reasons only partially understood, may, paradoxically, evoke typical acute inflammatory attacks of the disease [81, 82].

Treatment with anti-IL-1 agents, on the other hand, has been proven to be particularly efficacious in preventing attacks and inducing a rapid and long-lasting remission of the disease, as well as in the prevention and even regression of amyloidosis [77, 80, 83, 84].

Recently, the IL-6 receptor antagonist tocilizumab has been used in etanercept- and anakinra-resistant patients with good results, suggesting a possible role of IL-6 in the pathogenesis of TRAPS [85].

## 4. Cryopyrin-Associated Periodic Syndrome (CAPS)

Cryopyrin-associated periodic syndromes are a group of autoinflammatory diseases transmitted by autosomal dominant inheritance caused by mutations in the *NLRP3* gene (also called *CIAS1* or *PYPAF*) encoding for cryopyrin, a crucial inflammasome protein that directly activates IL-1*β*. To date, more than 90 *NLRP3* gene mutations have been identified, most of them in exon 3. These mutations induce an imbalance in IL-1*β* production, leading to fever attacks associated with other multiple inflammatory symptoms (Table 1) [16, 86, 87]. There are three known forms of CAPS. The least severe is familial cold autoinflammatory syndrome (FCAS) (OMIM 120100). Muckle-Wells syndrome (MWS) (OMIM 191900) is the clinical phenotype of medium severity. Finally, chronic infantile neurological cutaneous articular (CINCA) syndrome (OMIM 607115), also known as NOMID (neonatal-onset multisystem inflammatory disease), presents a decidedly more severe overall clinical picture [86, 87].

While FCAS and MWS may be family associated, CINCA syndrome—due to the seriousness of the clinical phenotype—is usually associated only with sporadic mutations [78, 88].

FCAS generally appears during the first few months of life and is characterized by brief recurrent inflammatory episodes, usually triggered by generalized exposition to low temperatures or sudden changes in temperature [89]. Recently, the possible emergence of a FCAS-like phenotype in adult patients or carriers of low-penetrance mutations has been described [90]. Symptoms include fever, urticaria-like rash that responds poorly to antihistamines, conjunctivitis, headache, arthralgia and/or arthritis, and fatigue. Generally, inflammatory attacks in FCAS decrease spontaneously [89], and an increase in acute-phase phlogistic indicators is usually seen during acute episodes [6]. Progress to amyloidosis is rather rare in patients with FCAS, in contrast with other CAPS [12, 89]. MWS is characterized by a variable clinical progression, with an episodic-recurrent or chronic pattern, and early childhood onset, usually in the first months of life. In addition to the symptoms typical of FCAS, patients often also manifest episcleritis, neurosensorial deafness, and secondary amyloidosis in up to 25% of cases [91, 92]. Finally, CINCA syndrome, the most severe of the CAPS, appears in the first weeks of life, being characterized by widespread nonpruritic urticaria-like skin rash [93–96]. In addition to the manifestations seen in FCAS and MWS, CINCA syndrome may also manifest with uveitis, papilledema, optic nerve atrophy leading to blindness, cerebral atrophy, mental retardation, increased intracranial pressure, ventriculomegaly, chronic aseptic meningitis, and, finally, a characteristic deforming osteoarthropathy of the large joints and hypertrophy of growth plates. Many patients present a typical *facies* characterized by prominent frontal eminences, saddle nose, and hypoplasia of facial bones. Lymphadenopathy and hepatosplenomegaly are also reported [93–96]. Also in CINCA syndrome, there is a risk of amyloidosis with frequent progressive kidney involvement [6]. In addition, from the laboratory point of view, all CAPS are characterized by persistent elevated neutrophil leukocytosis, increased acute-phase proteins, and chronic anemia [5, 6]. 

On the basis of etiopathogenetic mechanisms rooted in overproduction of IL-1*β*, CAPS have been treated with anti-IL-1 agents: anakinra was the first drug utilized in these patients, with exciting results from the neurological point of view as well [97, 98]. The safety and tolerability of rilonacept have been demonstrated in a group of pediatric and adult CAPS patients, while canakinumab has been shown to be safe and effective both in controlling clinical/laboratory indicators of disease activity and in controlling amyloidosis-related complications [14, 99–101].

## 5. Mevalonate Kinase Deficiency (MKD)

Also known as hyperimmunoglobulinemia D syndrome, MKD (OMIM 260920) is an autosomal recessive disease caused by mutations in the *MVK* gene [102] (Table 1), encoding for the enzyme mevalonate kinase, involved in the ATP-dependent phosphorylation of mevalonic acid into 5-phosphomevalonate. The most frequently found mutations are V377I, I268T, H20P/N, and P167L, at least one of which is found in 71.5% of patients [103]: they are responsible for reduced mevalonate kinase activity, which leads to overproduction of proinflammatory isoprenoids, reduced synthesis of cholesterol, and accumulation of mevalonic acid in plasma and urine [104]. The disease onset usually occurs during early childhood, generally within the first year of life, or in any case within the first 5 years. The emergence of symptoms after 5 years of age automatically excludes a diagnosis of MKD [105]. Acute episodes generally occur every 4–6 weeks and last about 3–7 days on average, with asymptomatic periods between attacks. The main clinical manifestations are recurrent fever (above 38.5°C), headache, mouth ulcers, abdominal pain, vomiting, and/or diarrhea (Table 2). More than 60% of patients may present with joint involvement in the form of arthralgia and/or arthritis, especially affecting large joints. During acute episodes, a nonspecific maculopapular rash may appear, while urticaria, erythema nodosum, and purpura are less frequently reported. Generalized lymphadenopathy, in particular, cervical, is very common among patients. Attacks are generally more frequent during childhood and adolescence, but the disease may persist into adulthood in more than half of patients [106]. Amyloidosis may be present in a smaller number of patients in comparison with other MAISs, estimated at around 3% of cases [106]. The possibility of macrophage activation syndrome during the course of an inflammatory attack has been observed in a patient with MKD [107].

A closely related disease is mevalonic aciduria (OMIM 610.377), which is due to a near-total inactivity of the enzyme mevalonate kinase: in this condition, recurrent fever episodes appear in association with serious systemic signs, such as delayed growth, cranial-facial dysmorphism, microcephalia, cerebellar atrophy, ataxia, psychomotor retardation, retinal dystrophy, and cataracts [108].

In terms of laboratory findings, MKD is invariably marked by leukocytosis and elevated phlogosis indicators during fever attacks, while many patients show increased serum IgD concentration (with levels above 100 IU/mL) and, less frequently, serum IgA between fever attacks. Urinary concentration of mevalonic acid may be increased during acute febrile flares and may thus sometimes be useful to the diagnosis [5, 6]. However, genetic testing to evaluate the *MVK* gene remains essential for a definite confirmation of MKD [6].

In terms of therapy, NSAIDs and corticosteroids may bring about partial relief of symptoms [109, 110]. Statins, in particular, simvastatin, seem efficacious in reducing the duration of acute episodes. The rationale behind their utilization is based on an attempt to reduce production of mevalonic acid by blocking the enzyme 3-hydroxy-3-methylglutaryl-coenzyme A reductase [111]. In resistant cases, treatment with anti-IL-1 [110, 112] and anti-TNF [110, 113] drugs has been proven to reduce both the frequency and the intensity of inflammatory attacks. 

## 6. NLRP12-Associated Autoinflammatory Disorder (NLRP12AD)

This is an autosomal dominant disease caused by mutations in the *NLRP12* gene encoding for the protein NLRP12 (or “monarch-1”), which plays a crucial role in immune system mechanisms against pathogenic agents (Table 1). As in the case of CAPS, it can be induced by generalized exposure to cold and is characterized by recurrent fever episodes lasting for 5–10 days accompanied by skin rash, headache, lymphadenopathy, mouth ulcers, and abdominal pain [114]. Treatment choice depends on the seriousness of the overall clinical picture and is based on the use of antihistamines, NSAIDs, and corticosteroids in less serious cases or the administration of anakinra in more serious ones [14, 114, 115]. However, loss of efficacy of anakinra has been described in a few patients [14, 116], raising the possibility of using anti-TNF-*α* and anti-IL-6 agents [116].

## 7. Granulomatous Autoinflammatory Diseases

Granulomatous autoinflammatory diseases include Blau syndrome (BS, OMIM 186580) and early-onset sarcoidosis (EOS, OMIM 609464); both are caused by mutations in the *NOD2/CARD15* gene, with subsequent dysregulation of the inflammatory response and formation of noncaseous granulomas (Table 1) [117]. 

Blau syndrome is an autosomal dominant granulomatous inflammatory disease caused by mutations in the region encoding for the nucleotide-binding domain region of the *NOD2/CARD15* gene (Table 1) [118, 119]: the protein NOD2 is mainly expressed in monocytes and plays a crucial role in the clearance of bacteria, particularly, *Mycobacterium tuberculosis*, as it is capable of interacting with peptidoglycan and activating the NF-*κ*B signal route [120]. 

The most frequently observed mutations are missense substitutions involving arginine residue at position 334 within the *NOD2/CARD15* gene (R334W or R334Q) [121, 122]. To date, this disease has been observed in about 200 patients. The onset is generally in childhood, around the age of 5, and the disease affects joints, skin, and eyes: the most common manifestation is a symmetrical polyarthritis of hands, feet, wrists, elbows, and ankles, which can also lead to joint ankylosis [123, 124]. Skin lesions may be in the form of dark red macular-papular-nodular rash or lichenoid-like lesions, which are generally symmetrical and appear on the trunk and/or limbs. Spontaneous healing may give rise to scarring. Under histological examination, the skin lesions present noncaseous granulomas with gigantic multinuclear cells [123, 124]. Eye involvement is the most serious complication of BS and is manifested in the form of recurrent bilateral anterior uveitis or bilateral granulomatous panuveitis, associated with eye pain, photophobia, and blurred vision. Ocular inflammation often leads to chorioretinitis, keratopathy, cataracts, glaucoma, or retinal detachment and may also involve other ocular structures such as conjunctiva, tear ducts, retina, and optic nerve. Additionally, BS has been described in association with persistent or intermittent fevers, granulomatous arteritis, cranial neuropathies, and hearing loss [123, 124].

The familial form of BS can be differentiated from EOS, a multiorgan sporadic disease characterized by onset in the first 4 years of life, joint, skin, eye, lymph node involvement, and recurrent fevers, with possible abdominal or central nervous system involvement. From the histological point of view, the presence of noncaseous epithelioid granulomas is observed in the involved tissues. Pulmonary parenchyma, involved in more than 90% of patients with adult sarcoidosis, is usually spared in EOS [125–127]. 

In spite of the notable clinical similarities, BS was initially considered a clinical entity distinct from EOS. Later, genetic analyses demonstrated that many patients with EOS also presented with mutations in the *NOD2/CARD15* gene. For this reason, some authors have proposed that BS and EOS are, respectively, familial and sporadic forms of the same disease [128]. Milman et al. recently proposed classifying patients with EOS as patients with “sporadic BS” in that they are carriers of mutations in the *NOD2/CARD15* gene and limiting diagnosis of EOS to those pediatric patients with sarcoidosis but no mutations [129].

There is no established therapy for patients with BS. In the acute phases, high doses of corticosteroids may be utilized with variable success [130, 131]. The use of anti-TNF-*α* and anti-IL-1 biological agents also seems encouraging [132–134].

## 8. Hereditary Pyogenic Disorders

The hereditary pyogenic disorders include PAPA (pyogenic arthritis, pyoderma gangrenosum, and acne) syndrome, Majeed syndrome (MS), and deficiency of the IL-1 receptor antagonist (DIRA): all of these disorders are characterized by the presence of sterile abscesses that mainly affect the skin, joints, and bones.

PAPA syndrome (OMIM 604416) is an autosomal dominant disease caused by mutations in the *PSTPIP1 *gene encoding for CD2-binding protein-1 (CD2BP1), involved in the proper assembly of the cytoskeleton, which normally inhibits pyrin-mediated inflammatory signals and the activation of caspase-1 [135–138]. It appears in early childhood and is characterized by joint involvement, manifested with severe self-limiting pyogenic arthritis. In terms of skin involvement, the appearance of pyoderma gangrenosum and nodular-cystic acne is described in early childhood [135]. Arthritic episodes usually respond readily to treatment with corticosteroids, while pyoderma gangrenosum is treated with topical immunosuppressant drugs [14, 134]. In a few reports, patients with PAPA syndrome responded wonderfully to treatment with anti-TNF-*α* and anti-IL-1 agents [139–141].

Majeed syndrome (OMIM 609628) is a very rare autosomal recessive disease described for the first and only time in 1989 in two brothers and a cousin with childhood-onset recurrent chronic multifocal osteomyelitis and congenital dyserythropoietic anemia; neutrophilic dermatosis was also reported in the two brothers [142]. The disease is caused by homozygous mutations in the *LPIN2* gene (Table 1) encoding for lipin 2, the role of which has not yet been clarified [143]. Clinically, this syndrome is characterized by recurrent fever attacks associated with multifocal sterile osteomyelitis, dyserythropoietic anemia, and chronic diffuse neutrophilic dermatosis with onset in early childhood [142]. Its treatment is empirically based on the use of NSAIDs and corticosteroids, although excellent results have recently been described with administration of anakinra and canakinumab [144, 145].

DIRA (OMIM 612852) is an autosomal recessive disease caused by missense mutations in the *IL1RN *gene encoding for the IL-1 receptor antagonist 1; there is a lack of endogenous self-regulation of IL-1 activity, with consequent excessive proinflammatory action of IL-1 itself [146] (Table 1). Disease onset is in the first weeks of life, and, in the initial phases, it may mimic neonatal sepsis, with multifocal osteomyelitis, periostitis, pustular skin lesions of various sizes, skin, ungueal alterations, hepatosplenomegaly, and the risk of multiorgan failure. Fever is generally not characteristic [146]. Radiological findings may include signs of osteolytic lesions, bone sclerosis, enlargement of the epiphysis of the long bones, and periosteal reaction [147, 148]. Laboratory findings include persistent elevation of acute-phase inflammatory indicators [146]. Due to the absence of endogenous IL-1 receptor antagonist, treatment is based on the use of anakinra, bringing about an excellent clinical improvement in a few days or weeks [146].

**Figure 1:**
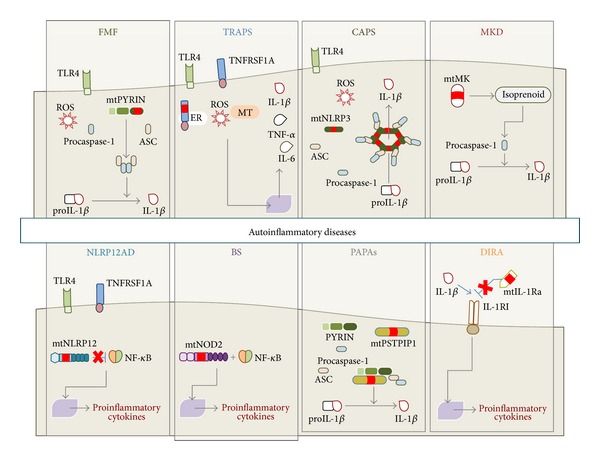
Schematic sketch showing the main pathophysiologic mechanisms of the monogenic autoinflammatory syndromes. Familial Mediterranean fever (FMF), cryopyrin-associated periodic syndromes (CAPS), mevalonate kinase deficiency (MKD), and PAPA syndrome (PAPAs) are due to mutations on pyrin (mtPYRIN), cryopyrin (mtNLRP3), mevalonate kinase enzyme (mtMK), and PSTPIP1 (mtPSTPIP1) proteins, respectively, and are associated with enhanced procaspase-1 activation, leading to increased IL-1*β* processing and secretion. Mutations in TNF receptors (TNFRSF1A) are responsible for tumor necrosis factor receptor-associated periodic syndrome (TRAPS). Indeed, it is known that intracellular accumulation of mutated TNFRSF1A (mtTNFRSF1A) in the endoplasmic reticulum (ER) enhances inflammatory responses. This condition leads to the activation of ER-stress response and mitochondria (MT) release of reactive oxygen species (ROS), which in turn promotes upregulation of proinflammatory cytokines, including IL-1*β*, TNF-*α*, and IL-6. NLRP12-associated autoinflammatory disorder (NLRP12AD) and Blau syndrome (BS) are related to mutated NLRP12 protein (mtNLRP12) and mutated NOD2 protein (mtNOD2), respectively, and they bring about nuclear factor-*κ*B (NF-*κ*B) deregulation. Deficiency of the interleukin-1 (IL-1) receptor antagonist (DIRA) is due to mutations on the gene coding for IL-1 receptor antagonist (IL-1Ra), which lead to loss of IL-1*β* inhibition and unopposed inflammatory burst. TLR4: toll-like receptor-4; ASC: apoptosis-associated speck-like protein containing a caspase recruitment domain; TNF-*α*: tumor necrosis factor-alpha;   IL-1*β*: interleukin-1*β*; IL-1Ra: interleukin-1 receptor antagonist; IL-1RI: IL-1 receptor type I; mtIL-1Ra: mutated IL-1Ra; IL-6: interleukin-6.

Thus, in conclusion, the elucidation of the molecular basis of MAISs has helped us recognize the consequences of excessive IL-1 signaling, proinflammatory isoprenoid production, or aberrant NK-*κ*B activation (Figure 1). Future studies will hopefully also evaluate the clinical benefit of different highly selective biologicals for each of the MAISs: the availability of these new therapeutic options for patients who have previously failed to respond to conventional treatments (NSAIDS, corticosteroids, colchicines, or immunomodulating agents) and the promise of patient-centered treatment strategies are doubtlessly the start of a new era in the management of these rare complex disorders.

## Figures and Tables

**Table 1:** Classification of the monogenic autoinflammatory syndromes.

	Inheritance	Gene	Chromosome	Mutated protein
Monogenic periodic fevers				
Familial Mediterranean fever (FMF)	AR	*MEFV *	16p13.3	Pyrin/marenostrin
Tumor necrosis factor receptor-associated periodic syndrome (TRAPS)	AD	*TNFRSF1A *	12p13	TNFRSF1A
Mevalonate kinase deficiency (MKD)	AR	*MVK *	12q24	Mevalonate kinase

Cryopyrin-associated periodic syndromes				
Familial cold autoinflammatory syndrome (FCAS)	AD	*NLRP3/CIAS1 *	1q44	Cryopyrin
Muckle-Wells syndrome (MWS)	AD			
Chronic infantile neurological cutaneous articular syndrome (CINCAs)	Sporadic, AD			
NLRP12-associated autoinflammatory disorder (NLRP12AD)	AD	*NLRP12 *	19q13.42	NLRP12 (monarch-1)

Autoinflammatory granulomatous disorders				
Blau syndrome (BS)	AD	*NOD2/CARD15 *	16q12	NOD2 (CARD15)
Early-onset sarcoidosis (EOS)	Sporadic	*NOD2/CARD15 *	16q12	NOD2 (CARD15)

Autoinflammatory pyogenic disorders				
Pyogenic arthritis pyoderma gangrenosum and cystic acne syndrome (PAPAs)	AD	*PSTPIP1 (CD2BP1) *	15q24-q25.1	PSTPIP1 (CD2BP1)
Majeed syndrome (MS)	AR, sporadic	*LPIN2 *	18q21.3-18q22	Lipin-2
Deficiency of the interleukin-1 receptor antagonist (DIRA)	AR	*IL1RN *	2q14	Interleukin-1 receptor antagonist

**Table 2:** Clinical, laboratory, genetic, and therapeutic aspects of the monogenic autoinflammatory syndromes.

	Onset age	Criteria and main suggestive clinical features	Laboratory findings	Therapy
FMF	First two decades of life and more rarely adulthood	*Tel-Hashomer diagnostic criteria.* (A) Major: (1) recurrent febrile episodes with serositis (peritonitis, pleurisy, and pericarditis) or synovitis; (2) AA amyloidosis in the absence of another predisposing disease; (3) good clinical response to daily administration of colchicine. (B) Minor: (1) recurrent febrile episodes; (2) erysipelas-like rash; (3) positive FMF family history in a first-degree relative. FMF diagnosis can be formulated on the basis of the presence of two major criteria or one major criterion and two minor ones; presence of one major and one minor criterion can point towards a probable FMF diagnosis. Secondary amyloidosis is not a rare event in patients not adequately treated or noncompliant patients.	Increased inflammatory markers (ESR, CRP, SAA, aptoglobin, and fibrinogen) Neutrophilic leukocytosis, anemia, thrombocythemia, and increased serum IgA and IgD levels; renal function tests and proteinuria/24 hours are needed (abnormal results can point towards secondary amyloidosis) *MEFV* analysis	Colchicine Anti-IL-1*β* agents Anti-TNF-*α* agents

TRAPS	Childhood and adolescence; adulthood	Recurrent inflammatory attacks (mean duration: 1–3 weeks) characterized by the following: fever, arthralgia; more rarely monooligoarthritis and/or tenosynovitis; periorbital edema, often associated with painful conjunctivitis, serpiginous erythematosus skin rash (migratory erythematous macules and/or painful plaques); polyserositis; abdominal pain, vomiting, diarrhoea, and constipation; chronic monocytic fasciitis with cramps and migratory myalgia; lymphadenopathy; headache, and fatigue, generalized malaise. Secondary amyloidosis is not rare.	Neutrophilic leukocytosis and thrombocythemia; renal function tests and proteinuria/24 hours are needed (abnormal results can point towards secondary amyloidosis) *TNFRSF1A* analysis	Corticosteroids Anti-IL-1*β* agents Anti-TNF-*α* agents

CAPS	First 6 months of life; in rare cases in adulthood	Fever, urticaria-like rash, conjunctivitis, and arthralgia.	Neutrophilic leukocytosis and thrombocythemia; increased inflammatory markers (ESR, CRP, SAA, aptoglobin, and fibrinogen); renal function tests and proteinuria/24 hours are needed (abnormal results can point towards secondary amyloidosis) *NLRP3* and * NLP12* analysis	Anti-IL-1*β* agents
	First months of life	Fever, urticaria-like rash, conjunctivitis, episcleritis, arthralgia, sensorineural hearing loss, and AA amyloidosis.		
	Perinatal onset	Fever, urticaria-like rash, anterior chronic uveitis, papillitis, optic nerve atrophy, arthralgia, chronic aseptic meningitis, sensorineural hearing loss, and AA amyloidosis.		

Mevalonate kinase deficiency	Early childhood	Inflammatory recurrent attacks (mean duration of 3–7 days) characterized by the following: fever; gastrointestinal involvement (abdominal pain, vomiting, and/or diarrhoea); polymorphic skin rash; painful lymphadenopathy, mainly laterocervical; splenomegaly; arthralgia and/or arthritis; headache, fatigue, and generalized malaise. Secondary amyloidosis is a rare but possible event.	Leukocytosis; increased inflammatory markers (ESR, CRP, SAA, aptoglobin, and fibrinogen); possible increase of serum IgD level (>100 IU/mL) in any phase of the disease; increased urinary levels of mevalonic acid during febrile attacks *MVK* analysis	NSAIDs and corticosteroids (with partial remission of acute symptoms) Anti-IL-1*β* agents Anti-TNF-*α* agents

Granulomatous disorders	Childhood	BS: triad of granulomatous arthritis, dermatitis, uveitis. EOS: polyarthritis with ocular, cutaneous, and lymph node involvement. Histologic findings are suggestive of noncaseating granulomatous inflammation.	Increased inflammatory markers (ESR and CRP) *NOD2/CARD15 *analysis	Corticosteroids Anti-IL-1*β* agents Anti-TNF-*α* agents

Autoinflammatory pyogenic disorders	Early childhood	PAPAs: recurrent self-limited sterile pyogenic arthritis, pyoderma gangrenosum, and nodulocystic acne. MS: multifocal sterile osteomyelitis, dyserythropoietic anemia, and chronic neutrophilic dermatosis. DIRA: multifocal sterile osteomyelitis, periostitis, pustulosis with ichthyosis-like features, nail alterations, and risk of multiorgan failure.	Increased inflammatory markers (ESR and CRP) *PSTPIP1, LPIN2,* and* IL1RN* analysis	Corticosteroids Anti-IL-1*β* agents (DIRA) Anti-TNF-*α* agents (PAPAs)

AD: autosomal dominant; AR: autosomal recessive; BS: Blau syndrome; CAPS: cryopyrin-associated periodic syndromes; CARD: caspase recruitment domains; CD2BP1: CD2-binding protein-1; CIAS1: cold-induced autoinflammatory syndrome-1; CINCAs: chronic infantile neurological cutaneous articular syndrome; CRP: C-reactive protein; DIRA: deficiency of the interleukin-1 receptor antagonist; EOS: early-onset sarcoidosis; ESR: erythrosedimentation rate; FCAS: familial cold autoinflammatory syndrome; FMF: familial Mediterranean fever; IL1RN: interleukin-1 receptor antagonist; LPIN2: lipin-2; MEFV: MEditerranean FeVer; MVK: mevalonate kinase gene; MKD: mevalonate kinase deficiency; MS: Majeed syndrome; MWS: Muckle-Wells syndrome; NLRP3: nucleotide-binding domain, leucine-rich repeat, and pyrin domain containing protein-3; NLRP12: nucleotide-binding domain, leucine-rich repeat, and pyrin domain containing protein-12; NOD: nucleotide-binding oligomerization domain; PAPAs: pyogenic arthritis with pyoderma gangrenosum and cystic acne syndrome; PSTPIP1: proline serine threonine phosphatase-interacting protein-1; SAA: serum amyloid-A; TNFRSF1A: TNF-receptor superfamily 1A; TRAPS: tumor necrosis factor receptor-associated periodic syndrome.
